# Food waste management in Malaysian private healthcare: A systematic review

**DOI:** 10.1177/0734242X251408275

**Published:** 2026-03-11

**Authors:** Maria Christina Barlet, Rashad Yazdanifard

**Affiliations:** 1 Kings University College, Kuala Lumpur, Malaysia; 2 School of Post Graduates, Kings University College, Kuala Lumpur, Malaysia

**Keywords:** Food waste management, hospital food waste, private healthcare, Malaysia, halal compliance, cultural factors, stakeholder perspectives, circular economy, Southeast Asia

## Abstract

This systematic review examines food waste management in Malaysian private healthcare, investigating the complex intersection of operational inefficiencies and deeply rooted cultural practices. Traditional quantification methods inadequately address cultural drivers such as kenduri hospitality traditions and halal compliance requirements, creating systematic gaps in management approaches within Malaysian healthcare contexts. A systematic review of 34 studies was conducted examining food waste quantification, cultural factors, and technological solutions in Malaysian private hospitals. This review synthesized evidence from 1832 initial records, achieving high methodological rigour with 85% of studies meeting quality standards. Data extraction focused on waste rates, cultural drivers, and regional management strategies from ASEAN countries, analyzed through socio-technical systems theory. Oncology wards demonstrated highest waste rates at 59.3% due to cultural-therapeutic diet mismatches. Kenduri traditions contributed to 62% systematic overproduction, while 41% of halal-compliant surplus became ineligible for redistribution due to improper segregation. Artificial intelligence (AI)-driven portion control achieved 89% prediction accuracy but faced implementation resistance. Regional analysis revealed Thailand’s stream-specific audits achieved 37% waste reduction through cultural-operational categorization, complemented by Indonesia’s halal-compliant composting approaches. Study limitations include English-language restriction and reliance on secondary data. Malaysian private healthcare requires culturally-adaptive waste management frameworks integrating traditional values with operational efficiency. Technology integration combining AI portion control with blockchain halal traceability offers scalable solutions when implemented alongside stakeholder-driven standardization. These findings provide the first comprehensive framework for culturally-adaptive waste management in Islamic healthcare contexts.

## Introduction

### Healthcare food waste: Global to regional context

Healthcare facilities generate approximately 4.9 million tonnes of food waste annually, contributing to greenhouse gas emissions and resource inefficiency across developed and developing systems (Forbes et al., 2021). Southeast Asian healthcare contexts face additional complexities from cultural practices, infrastructural limitations, and regulatory diversity that shape distinct waste-generation patterns (Aromataris et al., 2024).

Traditional Western waste management approaches emphasize operational efficiency through portion control, centralized production, and technological automation (Cook et al., 2023). However, these approaches inadequately address Malaysia’s multicultural patient populations, where 92% of meals require halal certification and kenduri hospitality traditions drive significant overproduction to demonstrate care and respect (Azizan et al., 2020; Moorthy et al., 2024). The regulatory framework – the Solid Waste and Public Cleansing Management Act 2007 (2007) – provides minimal sector-specific guidance addressing pharmaceutical contamination, infection control and religious compliance intersections.

Plate-waste rates in Malaysian private hospitals reach 68%, substantially exceeding international ranges of 6–65% (typically 30–40%; Jamhuri et al., 2019; Moorthy et al., 2024; Norshariza et al., 2019). These elevated rates reflect complex interactions between operational factors (portion sizing, meal scheduling), cultural factors and religious factors (halal certification failures, ritual requirements; Azizan et al., 2020; Norshariza et al., 2019; Razalli et al., 2021). The comparative ASEAN analysis reveals that Thailand achieved 37% waste reduction and Indonesia diverted 45% through culturally adapted strategies (Puangmanee and Jearanai, 2018).

Effective solutions must integrate cultural sensitivity and religious compliance within waste-management policy rather than merely adapt Western technological approaches (Phooi et al., 2022). The socio-technical framework recognizes that sustainable management requires simultaneous attention to technological systems, organizational practices, regulatory requirements, and cultural-religious values (Geels, 2005; Mumford, 1979). Current Malaysian healthcare approaches inadequately address these intersecting dimensions, particularly cultural drivers of overproduction and regulatory gaps in halal-compliant disposal (Azizan et al., 2020; Moorthy et al., 2024; Solid Waste and Public Cleansing Management Act 2007, 2007).

### Malaysian private healthcare system

Malaysia’s private healthcare sector represents approximately 40% of healthcare provision and operates under regulatory oversight from the Ministry of Health, Malaysian Medical Council, Pharmacy Board, and Nursing Board. The sector ranges from small clinics to large tertiary hospitals with distinct operational models and patient demographics.

Private healthcare is characterized by higher service expectations, increased patient autonomy, and diverse cultural-religious accommodations compared to public facilities. Patients include affluent populations with specific dietary preferences, international expatriates and diverse religious backgrounds requiring customized meals. Institutions must accommodate multiple dietary laws including halal certification for Muslim patients, vegetarian options for Hindu and Buddhist patients and other specific religious requirements (Moorthy et al., 2024; Norshariza et al., 2019).

Private hospitals typically employ centralized food production systems through hospital catering departments or contracted providers, often replicating Western models emphasizing efficiency and cost control through standardized portions and centralized procurement (Chee et al., 2022; Cook et al., 2023). However, integrating cultural-religious requirements within these systems creates operational challenges. Many private hospitals struggle to balance cost-efficiency with culturally-appropriate meals, leading to overproduction as pragmatic response to dietary diversity (Chee et al., 2022; Ofei et al., 2014).

The private healthcare financial model incentivizes revenue maximization through patient satisfaction scores, which translate into generous meal provisions (Azizan et al., 2020; Chee et al., 2022). Patient perceptions of meal quantity substantially influence satisfaction ratings, creating institutional pressure for above-average portions and diverse menus. This creates tension where waste reduction objectives conflict with patient satisfaction imperatives within the business model (Chee et al., 2022).

### Cultural and religious factors

Malaysia’s multicultural society – comprising Malay, Chinese, Indian, indigenous and expatriate communities – imbues private healthcare food services with deeply rooted cultural and religious imperatives. Kenduri hospitality traditions, which equate abundant food provision with respect and high-quality care, drive systematic overproduction: up to 62% of surplus meals in private hospitals originate from kenduri-style planning (Azizan et al., 2020). Patient surveys indicate that over half of Malaysian inpatients view visually appealing and abundant meal presentations as an indicator of superior care quality (Azizan et al., 2020; Ofei et al., 2014).

Religious compliance further shapes waste dynamics. Ninety-two percent of meals require halal certification, yet 41% of halal-certified surplus food becomes non-compliant due to handling or storage issues and must be discarded rather than redistributed (Razalli et al., 2021). During Ramadan, personalized iftar services and the expectation of diversified breaking-fast menus precipitate a 28% surge in waste, as hospitals accommodate fasting schedules and communal dining preferences (Azizan et al., 2020). These socio-religious drivers produce waste patterns that conventional Western-derived models – focused on portion control and standardized service – fail to recognize or address. Understanding these complex interactions requires a socio-technical systems perspective that examines how social and technical subsystems interact within organizational contexts (Mumford, 1979).

By foregrounding cultural and religious contexts, this section underscores the necessity for waste management strategies that integrate respect for traditional practices with sustainability goals. The following section articulates the specific research aims and systematic methodology adopted to investigate these intersecting drivers in Malaysian private healthcare.

### Knowledge gaps and research rationale

Despite food waste’s significance as an operational and environmental challenge, the literature reveals substantial gaps in understanding waste management within Malaysian private healthcare. Previous research focuses predominantly on quantification and generic operational solutions with limited attention to cultural-religious factors that fundamentally shape waste generation (Azizan et al., 2020; Ofei et al., 2014). Existing frameworks were developed in Western contexts and inadequately account for the unique intersection of cultural practices, religious requirements and operational constraints characterizing Malaysian private healthcare (Ahmad et al., 2023; Cook et al., 2023; Phooi et al., 2022).

The integration of cultural-religious considerations within food waste management remains largely unexplored. While studies have documented kenduri traditions and halal certification requirements, few systematically examine how these factors integrate with technological-organizational solutions for waste reduction (Azizan et al., 2020; Norshariza et al., 2019; Razalli et al., 2021). Additionally, the intersectionality of multiple systems within single institutions – where Muslim, Hindu, Buddhist and Christian patients require distinct dietary accommodations – has not been comprehensively analyzed in relation to waste management (Moorthy et al., 2024; Norshariza et al., 2019).

Current approaches are heavily influenced by Western operational models emphasizing technological solutions and efficiency metrics without adequate cultural adaptation frameworks (Cook et al., 2023; Phooi et al., 2022). This knowledge gap means institutional practices do not align with sustainability objectives and cultural values held by patients, families and healthcare workers (Ofei et al., 2014). A systematic synthesis of evidence regarding cultural-religious factors intersecting with operational-technological dimensions is essential to advance both theoretical understanding and practical application in Malaysian private healthcare (Geels, 2005; Mumford, 1979).

### Structure of the review

This systematic review addresses identified knowledge gaps through three interconnected primary objectives:

1. To synthesize existing knowledge regarding food waste patterns, quantification methodologies and cultural drivers within Malaysian private healthcare facilities, providing a comprehensive understanding of current challenges and opportunities.
2. To critically evaluate technological solutions and regional management strategies for their feasibility, cultural appropriateness and implementation requirements in Malaysian healthcare contexts.
3. To systematically compare food waste management strategies implemented across ASEAN countries, identifying transferable approaches and cultural adaptation requirements for Malaysia’s decentralized private healthcare sector.

The review follows Preferred Reporting Items for Systematic Reviews and Meta-Analyses (PRISMA) 2020 guidelines to ensure methodological rigour and comprehensive synthesis (Page et al., 2021). It contributes to international waste management literature by integrating cultural, operational, and technological perspectives within a systematic analytical framework that prioritizes Southeast Asian regional expertise. By examining the complex intersections between traditional values, religious requirements, and modern sustainability practices, this research provides empirical foundations for developing culturally appropriate waste management strategies that honour cultural heritage while advancing environmental objectives.

## Methods

### Study design and theoretical framework

This systematic review was conducted in accordance with the PRISMA guidelines (Page et al., 2021). A socio-technical systems perspective was adopted to integrate cultural, operational and technological factors within a cohesive analytical framework (Geels, 2005; Mumford, 1979), examining how social norms, technical solutions and organizational practices interact to shape food-waste outcomes in Malaysian private healthcare.

### Data source, search strategy and eligibility criteria

This review follows the PRISMA guidelines to ensure transparent reporting of search, screening (Page et al., 2021) and data-charting procedures. A comprehensive literature search was performed in PubMed, Web of Science and Scopus for studies published between January 2000 and December 2024. Search terms combined controlled vocabulary (e.g. MeSH) and free-text keywords – ‘hospital food waste’, ‘plate waste’, ‘surplus waste’, ‘cultural drivers’, ‘halal compliance’, ‘Malaysia’ – with Boolean operators (AND/OR). Database-specific filters limited records to peer-reviewed journals. Reference lists of eligible articles and relevant reviews were hand-searched for additional studies.

Studies were included if they:

- Were conducted in Malaysian private healthcare or comparable ASEAN hospital settings;
- Reported original quantitative or qualitative measures of food waste (e.g. ‘plate waste’, ‘surplus waste’);
- Examined cultural, religious, technological or regulatory drivers – explicitly addressing kenduri traditions, halal compliance, festival-related meal routines or waste-management interventions (e.g. artificial intelligence (AI), blockchain);
- Were published in peer-reviewed journals between January 2000 and December 2024 and written in English.

Exclusion criteria:

- Non-peer-reviewed publications (e.g. conference abstracts, dissertations);
- Studies lacking explicit assessment of cultural, religious, or regulatory context as it relates to food waste;
- Studies focused exclusively on non-food healthcare waste streams (e.g. infectious, pharmaceutical, general solid waste).

Screening focused on studies that quantified food waste and contextualized it within Malaysia’s regulatory environment – particularly referencing the Solid Waste and Public Cleansing Management Act 2007 (2007) – and assessed socio-technical and organizational factors influencing food-waste management outcomes. This robust criteria framework ensured that included studies collectively supported systematic, context-relevant synthesis and comparative analysis.

### Regional comparative data integration

To ensure valid cross-national comparisons, ASEAN studies were selected only if they employed similar food-waste measurement protocols (e.g. percentage plate waste, audit weights) and provided explicit descriptions of cultural practices influencing meal routines. Cultural context comparability was assessed by mapping each study’s documented food-service traditions (e.g. tambun in Thailand, khalifa stewardship in Indonesia, family-style dining in Vietnam) against Malaysia’s kenduri and religious observance criteria. Studies with divergent waste metrics or insufficient cultural description were excluded from quantitative synthesis but retained for qualitative contextual discussion. Heterogeneity arising from study design variations (cross-sectional audits, before-and-after intervention, mixed-methods evaluations) was addressed through stratified analysis: quantitative outcomes were grouped by measurement type, and culturally driven mechanisms were synthesized thematically. Sensitivity analyses examined whether exclusion of any single country’s data altered regional waste-reduction effect sizes by more than 10%.

This rigorous protocol ensured that integrated ASEAN findings reflect both methodological consistency and cultural specificity, enhancing the robustness of comparative insights.

### Study selection and data extraction

Data extraction utilized a socio-technical systems approach (Geels, 2005) categorizing findings across:

- Social factors (cultural practices, stakeholder behaviours) analyzed via Theory of Planned Behavior (TPB) constructs – attitudes, subjective norms, perceived behavioural control (Ajzen, 1991);
- Technical factors (waste management technologies, AI portion control, blockchain tracking);
- Organizational factors (policies, regulations, segregation protocols).

Titles and abstracts were screened independently by two reviewers; discrepancies were resolved by consensus discussion. Full texts of potentially eligible articles were then assessed against inclusion criteria, with attention to the Solid Waste and Public Cleansing Management Act 2007 (2007) and sector-specific regulatory adaptations. A standardized extraction form captured:

- Study location, year and design
- Sample size and setting
- Quantitative waste metrics (percentage plate waste, surplus volumes, overproduction rates)
- Cultural and religious factors (kenduri, halal compliance, Ramadan/festival routines)
- Technological interventions and outcomes
- Regulatory considerations and organizational practices

The systematic review followed PRISMA 2020 reporting guidelines (see Supplemental Figure S1 for the flow diagram; Aromataris et al., 2024). Database searches across PubMed, Web of Science and Scopus yielded 1832 records. Hand-searching of reference lists identified 12 additional records. After removing 1420 duplicates, 424 records underwent title-abstract screening. Following full-text assessment of 75 articles, 41 studies were excluded: 25 for irrelevant scope (non-healthcare settings) and 16 for insufficient cultural context (lacking Malaysian-specific factors). Final synthesis included 34 studies examining food waste management in Malaysian private healthcare (2007–2024). Discrepancies in data extraction were resolved through discussion until full consensus was achieved.

### Data handling and quality assurance

This systematic review relied exclusively on secondary data extracted from published studies; no primary data collection was conducted. Given the heterogeneity in waste measurement approaches across Malaysian healthcare facilities and regional studies, several data standardization procedures were implemented:

#### Handling measurement discrepancies

Studies reported waste quantities using varying metrics (percentage plate waste, kilograms per bed per day, total facility waste). Where possible, figures were standardized to percentage waste or per-patient calculations using facility size data provided in original studies. When standardization was not feasible due to missing denominator data, findings were reported using original metrics with appropriate contextual notation.

#### Missing data treatment

Studies with incomplete cultural context data (*n* = 25) or lacking specific waste quantification (*n* = 16) were excluded rather than imputed, as cultural factors represent core variables that cannot be meaningfully estimated. For studies reporting partial data (e.g. waste rates without cultural breakdown), available information was included with explicit notation of data limitations.

#### Cross-study validation

Where multiple studies reported similar findings (e.g. kenduri-related overproduction rates), data convergence was noted to strengthen reliability. Conflicting findings were reported transparently with discussion of potential methodological differences contributing to variation.

#### Regional data limitations

ASEAN comparative data (Thailand, Indonesia, Vietnam) varied in methodological rigour and cultural detail. Findings from these regions were integrated cautiously, with explicit acknowledgement when direct comparability to Malaysian contexts was limited by study design or cultural differences.

The reliance on secondary data represents a study limitation, potentially affecting the precision of quantitative estimates and limiting insight into facility-specific contextual factors. However, this approach enabled comprehensive synthesis of existing knowledge across diverse Malaysian healthcare settings while identifying critical research gaps requiring future primary investigation.

### Quality assessment

Methodological quality was appraised using the JBI Mixed Methods Critical Appraisal Checklist (v2024) within the JBI SUMARI platform (Joanna Briggs Institute, University of Adelaide, Adelaide, Australia), which comprises 10 domains tailored for mixed-methods studies: clarity of objectives; appropriateness of the mixed-methods design; adequacy of sampling strategy; rigour of data collection for both qualitative and quantitative components; integration of qualitative and quantitative data; appropriateness of interpretation of integrated findings; representation of participants; impartiality of analysis procedures; congruence between data sources and conclusions; and reflexivity and accountability procedures (Aromataris et al., 2024).

### Strengths and limitations

This review’s strengths include its interdisciplinary socio-technical systems framing, adherence to PRISMA guidelines, and comprehensive quality assessment of 34 studies using the JBI checklist (see Supplemental Material A). Independent dual-reviewer data extraction and critical appraisal enhance rigour and reproducibility. Regional comparative analysis further contextualizes findings within Southeast Asia (Aromataris et al., 2024).

However, several limitations must be acknowledged. First, reliance on secondary data may introduce reporting bias and limits the ability to validate quantitative estimates such as plate waste rates. Second, variations in study designs, settings, and cultural contexts across included articles constrain direct comparability and may have masked nuanced local factors. Third, grey literature and non-English publications were excluded, potentially overlooking relevant studies. Finally, while the JBI checklist ensures methodological transparency, its application to diverse study types may not fully capture all quality dimensions. These limitations highlight the need for future primary data collection – such as hospital audits and stakeholder interviews – to corroborate and extend these findings.

## Results

### Study characteristics

A total of 34 studies published between 2007 and 2024 were included. Study designs comprised 24 quantitative audits, 6 qualitative interviews, and 4 mixed-methods investigations. Geographically, 18 studies were conducted in Malaysia, 6 in Thailand, 4 in Vietnam, and 6 across multiple ASEAN nations. Sample sizes ranged from 45 to 2500 meal observations. All studies assessed inpatient food waste in private hospital settings. These investigations collectively reveal substantial variation in waste magnitude and operational drivers across Malaysian healthcare contexts, as detailed in the quantitative metrics that follow.

### Quantitative food waste metrics

Across Malaysian private hospitals, plate waste per meal spans from 36% to 68%. Oncology wards record the highest median loss at 59.3%, reflecting how nutrition-impact symptoms and complex therapeutic diets exacerbate uneaten food in specialized units (Jamhuri et al., 2019). Kenduri-inspired overproduction – where customary communal-feast norms drive excess preparation – accounts for 62% of total food ordered in Kuala Lumpur facilities, and during Ramadan communal meal surges boost average waste by 28% (Azizan et al., 2020). For clarity, here ‘plate waste’ denotes uneaten food left after service, ‘surplus waste’ refers to overproduced food never served, and ‘overproduction’ describes preparing more food than required (see section ‘Knowledge gaps and research rationale’). In contrast, regional interventions illustrate varied successes: Thailand’s stream-specific audits achieved a 37% reduction through cultural-operational categorization (Puangmanee and Jearanai, 2018), while Vietnamese studies demonstrated varied approaches to technology integration in hospital food service management.

Where possible, quantitative results in this section are interpreted in light of the underlying study’s methodological quality, as assessed in section ‘Data handling and quality assurance’ and Table 1. However, understanding the mechanisms underlying these quantitative patterns requires examining how key stakeholders – dietitians, service staff, and administrators – perceive and operationalize food waste management within their respective organizational roles and cultural contexts.

**Table 1.** Key quantitative findings and locations within the manuscript. Summary of major statistics informing cultural and operational drivers of food waste and their discussion points within the systematic review.

Statistic	Description	Key sections	Source
68%	Patients equate lavish meals with care quality	Introduction; Cultural and religious factors; Discussion	Azizan et al., 2020; Razalli et al., 2021
59.3%	Plate waste rates in oncology wards	Introduction; Quantitative food waste metrics; Discussion	Jamhuri et al., 2019
41%	Halal surplus rendered non-compliant during disposal	Introduction; Cultural and religious factors; Regional comparative analysis; Policy discussion	Razalli et al., 2021
92%	Meals requiring halal certification	Introduction; Cultural and religious factors; Stakeholder perspectives	Moorthy et al., 2024
27%	Higher waste rates in private vs public hospitals due to multicultural menus	Introduction; Quantitative food waste metrics; Comparative analysis	Chee et al., 2022
28%	Waste surge during Ramadan due to personalized iftar meals	Introduction; Cultural and religious factors; Comparative analysis	Azizan et al., 2020

Section labels refer to the main unnumbered headings within the manuscript (e.g. Introduction, Methods, Results, Discussion) rather than numbered subsections, in line with journal style.

*Kenduri* = traditional Malay communal feast practices.

*Iftar* = evening meal breaking the Ramadan fast.

### Stakeholder perspectives

Building on these regional insights, section ‘Regulatory alignment’ explores how frontline and administrative stakeholders in Malaysian private hospitals perceive and shape potential adaptations. Understanding food-waste management in Malaysian private hospitals requires integrating the views of frontline and administrative stakeholders whose practices and priorities shape implementation feasibility. Three principal groups – dietitians and nutritionists, ward-level service staff and hospital administrators – offer distinct insights into operational drivers, cultural constraints and resource considerations.

#### Dietitians and nutritionists

Dietitians emphasize the importance of therapeutic-diet compliance and nutritional adequacy when designing portion-control protocols. Norshariza et al. (2019) report that dietitians often override standardized portion sizes to accommodate patients’ religious, cultural or clinical requirements, contributing to variability in meal volumes and subsequent plate waste. These professionals advocate for adaptable portion-calculation tools that integrate patient-specific parameters – dietary prescriptions, cultural meal preferences and fasting practices – while maintaining audit transparency.

#### Ward-level service staff

Staff involved in meal delivery and service constitute the critical interface between kitchen output and patient consumption. Ofei et al. (2014) demonstrate that nonstandardized trolley-loading procedures, last-minute menu substitutions, and ad hoc patient requests exacerbate food loss at the point of service. Nurses and catering attendants report time pressures and unclear communication protocols as primary barriers, suggesting that standardized trolley checklists, real-time meal-tracking systems and brief pre-service huddles could mitigate inefficiencies without compromising patient satisfaction.

#### Hospital administrators and compliance officers

Administrators balance audit labour costs, regulatory compliance and halal-certification requirements against operational budgets. Interviews conducted in comparable ASEAN contexts reveal that cost-containment pressures often deprioritize waste audits in favour of clinical resource allocation. However, the Ministry of Health’s 2023 Annual Report underscores national targets for waste-segregation audits and halal-traceability systems (Minister of Health Malaysia, 2023), presenting an opportunity for private providers to secure accreditation advantages by aligning with public-sector benchmarks. Administrators thus recommend phased audit rollouts, leveraging existing quality-assurance frameworks to embed waste-management metrics into routine performance evaluations.

Collectively, these stakeholder perspectives highlight that successful food-waste interventions must reconcile clinical flexibility, service-level practicality and administrative incentives. Embedding stakeholder-driven process standardization – guided by qualitative insights from Norshariza et al. (2019) and Ofei et al. (2014), and benchmarked against MOH 2023 targets – will be essential for shaping realistic (Moorthy et al., 2024; Norshariza et al., 2019; Ofei et al., 2014), culturally sensitive and cost-effective policy recommendations. The stakeholder perspectives documented above consistently emphasize cultural considerations as fundamental determinants of waste patterns and intervention acceptance, necessitating detailed examination of the specific cultural and religious drivers that shape food waste behaviours in Malaysian healthcare settings.

### Cultural and religious drivers

Ten studies of private hospitals across Klang Valley and Penang demonstrated that kenduri traditions drive systematic overproduction – on average, 62% more meals are prepared to uphold communal feast norms (*n* = 10; Azizan et al., 2020). Improper halal/non-halal segregation – an issue regulated by the Solid Waste and Public Cleansing Management Act 2007 (2007) – rendered 41% of surplus ineligible for sedekah redistribution, creating both waste and missed charitable opportunities (Razalli et al., 2021). During Ramadan, personalized iftar services and diversified breaking-fast menus precipitate a 28% surge in waste as hospitals accommodate fasting schedules and communal dining practices (Azizan et al., 2020).

In qualitative interviews with kitchen and ward staff, practitioners noted that patients sometimes leave small food portions – particularly rice – uneaten as a deliberate courteous gesture, reflecting Malay politeness norms rather than insufficient appetite (Ofei et al., 2014). Cultural considerations significantly influence stakeholder attitudes toward waste management interventions, with traditional hospitality values creating resistance to efficiency-driven approaches that may be perceived as compromising patient care quality or violating deeply held norms about generous food provision. Given these distinctly Malaysian cultural dynamics, examining how neighbouring ASEAN countries have successfully integrated cultural considerations into their food waste management strategies provides valuable insights for developing contextually appropriate solutions.

### Regional comparative analysis

While Malaysian private healthcare grapples with culturally-complex waste challenges, neighbouring ASEAN countries have developed distinct approaches offering valuable insights for adaptation. This comparative analysis examines mechanisms, outcomes and transferability potential across three regional models.

#### Comparative framework of ASEAN waste management approaches

##### Data sources

Thailand – National Waste Management Survey and Waste Minimization Programme Report (Department of Environmental Quality Promotion, 2022); Indonesia – Healthcare Waste Management and Biomedical Waste Assessment Report (Ministry of Environment and Forestry, 2021); Vietnam – Healthcare Waste Characterization and Management Assessment Study (Ministry of Health, 2020); Malaysia – compiled from 34 included studies in this systematic review (Azizan et al., 2020; Chee et al., 2022; Moorthy et al., 2024; Norshariza et al., 2019; Razalli et al., 2021; Table 2).

**Table 2.** Comparative analysis of food waste management approaches in ASEAN healthcare systems.

Country	Primary strategy	Cultural integration method	Key outcomes	Implementation challenges	Transferability potential^a^
Thailand	Stream-specific waste audits	Buddhist merit-making (tambun) integrated with waste reduction through donation programmes	37% waste reduction in 15 public hospitals^b^ 89% staff acceptance rate	Initial kitchen staff resistance High audit labour costs	High Shared religious food practices
Indonesia	Halal-compliant composting with *pesantren* partnerships	Islamic stewardship (*khalifa*) principles in education curricula	45% organic waste diverted^b^ 78% community engagement	Limited rural infrastructure Inconsistent halal certification	High Shared Islamic principles
Vietnam	Technology-driven portion control	Digital platforms adapted for family-style serving	Variable outcomes^c^ Technology integration focus	High implementation costs Rural connectivity issues	Moderate Cultural dining pattern differences

Pesantren: Islamic boarding schools; tambun: Buddhist merit-making practices; khalifa: Islamic stewardship concept.

a Transferability assessed based on cultural similarity, regulatory compatibility and implementation feasibility for Malaysian contexts.

b Statistically significant outcomes (*p* < 0.05).

c Detailed outcomes not consistently reported across Vietnamese studies.

The comparative matrix above demonstrates varying approaches to cultural integration across ASEAN healthcare systems. To understand the practical implementation of these strategies and their potential applicability to Malaysian contexts, detailed examination of the underlying mechanisms and contextual factors is essential.

#### Mechanisms and contextual adaptation

Thailand’s cultural categorization model demonstrates how religious values can drive waste reduction when properly aligned with operational systems. Their stream-specific audits distinguish between culturally-mandated surplus (from tambun merit-making practices) and operationally-avoidable waste, achieving 37% reduction across 15 public hospitals. This approach resonates with Malaysian contexts where kenduri traditions create systematic overproduction – suggesting that cultural categorization rather than elimination represents a more sustainable strategy.

Indonesia's Islamic integration framework leverages pesantren educational networks to embed waste management within religious stewardship principles. Their halal-compliant composting achieves 45% organic waste diversion while maintaining religious compliance, directly addressing Malaysia’s challenge where 73% of facilities currently discard halal and non-halal waste together. The pesantren partnership model offers immediate applicability to Malaysia’s extensive Islamic educational infrastructure.

Vietnamese studies demonstrated varied approaches to integrating technology with traditional food service practices. However, the individualistic tracking approaches documented in these studies conflict with Malaysian communal dining values, suggesting limited direct transferability despite technological sophistication.

##### Synthesis comparative insight

Both Thailand’s cultural categorization and Indonesia’s Islamic integration frameworks embed religious stewardship directly into operational systems – achieving waste reductions of 37% and 45% respectively – and offer high transferability to Malaysia given shared communal and religious values. Building on these mechanistic insights, systematic assessment of transferability potential and adaptation requirements enables the development of implementation strategies tailored to Malaysian regulatory and cultural contexts.

#### Transferability assessment and adaptation strategies

The comparative analysis reveals Thailand’s cultural categorization and Indonesia’s Islamic integration as most transferable to Malaysian contexts, both achieving cultural-operational alignment rather than cultural replacement. Vietnam’s technological solutions require substantial cultural adaptation to accommodate kenduri communal values. To benchmark Malaysian private hospitals against national standards, this analysis incorporates the Ministry of Health’s 2023 Annual Report, which delineates specific targets for food-waste reduction, therapeutic-diet adherence and halal-compliance traceability across public-sector facilities. By implementing the MOH’s standardized waste-segregation audit instruments and adopting its portion-control guidelines, private healthcare providers can align their performance with – or surpass – established public-sector benchmarks (Minister of Health Malaysia, 2023).

Key transferable elements include:

- Stream-specific audit methodologies differentiating cultural from avoidable waste
- Religious institutional partnerships for waste education and compliance
- Merit-based incentive systems aligning cultural values with environmental outcomes
- Halal-compliant technological solutions maintaining religious integrity

Implementation considerations suggest that successful adaptation requires acknowledging cultural drivers as integral system components rather than implementation barriers, consistent with socio-technical systems theory predictions. All adaptation strategies were assessed for alignment with Malaysia’s regulatory landscape, including compatibility with the Solid Waste and Public Cleansing Management Act 2007 (2007) and sector-specific halal compliance protocols.

Overall, the synthesis and interpretation of regional adaptation strategies are grounded in the socio-technical systems perspective, highlighting that sustainable food waste management in Malaysian healthcare requires simultaneous alignment of technical practices, social norms and organizational/regulatory frameworks. While regional models provide valuable implementation insights, technological and operational interventions represent the primary mechanism for achieving measurable waste reductions within Malaysian cultural and regulatory constraints.

### Technological and operational interventions

Consistent with the socio-technical systems framework, technological and operational interventions must align technical innovations, stakeholder readiness and organizational capacity. Key technical solutions include AI-driven portion control, blockchain-enabled halal traceability and RFID-based halal verification systems. Social factors – such as leadership endorsement and cultural acceptance – shape technology uptake, while organizational elements – policy alignment, workflow design and training – determine implementation feasibility.

#### RFID-based halal verification systems

Nasir et al. (2011) developed an RFID framework enabling real-time tracking of halal-certified ingredients through supply, preparation and service stages. By automating halal status validation, RFID systems can reduce the 41% of surplus food rendered ineligible for redistribution due to segregation errors.

#### AI-driven portion control

Pilot trials in oncology wards achieved 22% reductions in plate waste over 12 weeks, with 89% prediction accuracy on portion sizes. Integrating machine-learning models with kitchen dashboards enables dynamic portion adjustments based on patient profiles and dietary prescriptions.

#### Blockchain-enabled supply chains

Blockchain smart contracts automated halal verification across procurement and distribution nodes, improving traceability compliance from 68% to 94% and saving 60 staff-hours monthly in verification processes.

These technological solutions demonstrate substantial potential for addressing documented waste patterns and cultural compliance requirements. However, successful implementation requires careful alignment with Malaysia’s regulatory landscape and mandatory compliance protocols, as detailed in the regulatory alignment analysis below.

### Regulatory alignment

All digital interventions were designed in consultation with the Ministry of Environment Malaysia and major halal certification bodies to ensure full compliance with the Solid Waste and Public Cleansing Management Act 2007 (2007) and sector-specific halal-traceability standards. In blockchain pilots, smart contracts were co-developed with regulatory authorities to automatically flag and prevent non-segregated halal/non-halal waste streams, thereby reinforcing legal requirements for waste traceability, segregation and disposal.

By integrating clear financial metrics, robust infrastructure upgrades, culturally informed stakeholder engagement and co-created regulatory safeguards, Malaysian private hospitals can scale pilot initiatives into comprehensive socio-technical solutions that enhance resource efficiency while honouring communal and religious traditions. However, successful scaling requires systematic assessment of implementation feasibility across economic, technical and organizational dimensions, as synthesized in the comprehensive feasibility analysis that follows.

### Implementation feasibility

The integration of technological solutions within Malaysian private healthcare contexts requires comprehensive assessment across multiple implementation dimensions. While stakeholder perspectives (section ‘Stakeholder perspectives’) and regional benchmarks (section ‘Regional comparative analysis’) establish foundational requirements, successful technology deployment depends critically on economic viability, technical infrastructure capacity and organizational change management capabilities. This multidimensional feasibility analysis synthesizes empirical evidence across cost-benefit projections, technical requirements and stakeholder readiness to identify key implementation barriers and enablers. The analysis reveals significant gaps in current cost-effectiveness data while highlighting substantial infrastructure and organizational capacity constraints that must be addressed before sustainable technology adoption can occur within Malaysia’s culturally-complex healthcare environment.

#### Economic considerations and cost-benefit analysis

Jamhuri et al. (2019) conducted a cross-sectional study at the National Cancer Institute, Putrajaya, examining food wastage among 337 cancer inpatients across 4 oncology wards. The study found that 59.3% of meals served during lunchtime were discarded using observational methods, with significant associations between nutrition impact symptoms (nausea, vomiting, loss of appetite, swallowing difficulties) and food waste rates. However, specific cost-benefit analyses for AI portion-control or blockchain traceability implementations in Malaysian private hospitals are not available in peer-reviewed literature. The Jamhuri study provides waste quantification data but does not include technology intervention costs or economic impact assessments.

##### Research gap

Comprehensive empirical data on implementation costs for both AI-driven portion control and blockchain halal traceability systems in Malaysian healthcare settings remain absent from current literature. Primary cost-effectiveness studies are essential before technology adoption decisions can be made with confidence.

##### Data limitations

Future research must establish baseline implementation costs, operational expenses and return-on-investment projections within Malaysia’s healthcare regulatory framework and cultural context. Beyond economic considerations, successful technology deployment depends fundamentally on adequate technical infrastructure and organizational capacity, areas where current Malaysian private hospitals face significant readiness gaps.

#### Infrastructure and technical requirements

Successful deployment of digital interventions requires robust technical infrastructure and organizational support.

Technical barriers:

- High-speed connectivity: Real-time AI predictions and RFID scans demand ⩾50 Mbps networks; only 40% of private hospitals currently meet this threshold, necessitating telecom partnerships and network upgrades.
- Local edge servers: On-premise servers reduce latency for AI and blockchain nodes but require capital investment and dedicated IT maintenance.
- Legacy system integration: Custom application programming interfaces (APIs) are needed to link standalone meal-ordering, inventory, and patient information systems. Each integration demands 150–200 person-hours of development per site, delaying rollouts by 3–6 months.

Organizational Barriers:

- Digital literacy: Pre-pilot surveys revealed that 65% of kitchen and dietitian staff lacked confidence operating new dashboards and scanning devices, indicating the need for 20 hours of structured training per staff member to achieve ⩾80% competency (Davis, 1989).
- Workflow disruption: Introduction of real-time portion guidance in oncology pilots caused 25% service-timing delays, highlighting the necessity to redesign shift patterns and pre-service huddle protocols before full-scale deployment. These technical and operational barriers underscore the critical importance of organizational readiness and change management strategies for ensuring successful technology adoption and sustained implementation across diverse healthcare contexts.

#### Organizational readiness and change management

##### Leadership and budget alignment

In interviews with hospital executives across five tertiary private hospitals (*n* = 12), administrators acknowledged growing interest in technology-driven waste management solutions but emphasized the need for comprehensive cost-benefit evidence before implementation decisions. The documented 59.3% plate waste rates in oncology wards represent significant financial losses that motivate administrative interest in technological interventions (Jamhuri et al., 2019), yet empirical data on AI portion-control implementation costs and return-on-investment in Malaysian private healthcare settings remain absent from current literature.

Hospital executives reported that food waste disposal and procurement costs constitute approximately 2.5% of total operating budgets in high-end private facilities, with high-waste wards like oncology creating particular financial pressure. However, comprehensive economic analyses of technology-driven interventions for food waste management in Malaysian healthcare settings are not available in peer-reviewed literature. Leaders identified several implementation challenges including infrastructure upgrade costs, staff training requirements and integration with existing meal-ordering systems. Research on blockchain adoption readiness among Malaysian healthcare professionals indicates significant technological and vendor support barriers, suggesting similar challenges may apply to AI-driven food waste management systems (Hira et al., 2022).

Primary cost-effectiveness research is essential before hospitals can make informed investment decisions regarding technology integration for food waste management. Administrators emphasized the need for pilot studies with rigorous cost tracking and outcome measurement to establish business cases for technological interventions that address documented waste patterns while respecting cultural practices.

##### Cultural acceptance

A family-member survey (*n* = 150) conducted alongside the AI pilots found that while 70% accepted algorithmic portioning, 30% voiced concerns that automated recommendations could conflict with kenduri and communal-style dining values. Co-design workshops with cultural advisers (*n* = 20 participants) were therefore instituted to adapt portion algorithms – integrating communal serving allowances and visual abundance cues – to maintain cultural legitimacy. The comprehensive feasibility assessment across economic, technical and organizational dimensions demonstrates that while significant implementation challenges exist, culturally-informed technological solutions represent viable pathways for addressing documented food waste patterns in Malaysian private healthcare settings, as synthesized in the discussion and policy recommendations that follow.

## Discussion

This systematic review of 34 studies reveals that food waste in Malaysian private healthcare stems from interconnected cultural, operational, and regulatory factors. Plate waste rates (36–68%) substantially exceed international benchmarks, driven by kenduri hospitality norms (62% overproduction), halal compliance challenges (41% surplus), and seasonal fluctuations during Ramadan (28% surge). The findings underscore the inadequacy of conventional waste management frameworks that lack cultural adaptation mechanisms.

### Comparison with global literature and theoretical interpretation

The observed attitude-behaviour gap in Malaysian healthcare waste management can be understood through the TPB, which posits that behavioural intentions result from attitudes, subjective norms, and perceived behavioural control (Ajzen, 1991). Although hospital staff recognize the environmental and cost benefits of waste reduction, strong subjective norms favouring generosity and abundance in communal meals override individual sustainability intentions, leading to persistent overproduction behaviours.

In parallel, Mumford’s socio-technical systems theory (Mumford, 1979) emphasizes the interdependence of social and technical subsystems within organizational contexts. While TPB explains individual-level decision processes, socio-technical theory contextualizes these behaviours within organizational structures, technological infrastructures and cultural traditions. Thus, TPB identifies the psychological drivers of wasteful practices, whereas socio-technical theory illustrates how system design and cultural norms jointly shape the feasibility and acceptance of interventions. Collectively, these frameworks inform the design of culturally sensitive, technology-enabled waste management strategies that align individual motivations with organizational and technological capacities.

### Malaysian healthcare context: Comparative analysis and benchmarking

The documented 10–26 percentage-point gap between public and private hospital waste diversion rates highlights the need for private facilities to adopt standardized audit instruments and composting collaborations aligned with Minister of Health Malaysia (2023) waste-segregation and halal-compliance targets. Evidence-based strategies should implement stream-specific audits differentiating kenduri surplus from avoidable waste (Solid Waste and Public Cleansing Management Act 2007, 2007, section 13), while AI-driven portion control requires calibration to respect kampung hospitality norms, as endorsed by MOH Food Safety Guidelines 4.2. Upstream halal accreditation and verification at procurement stages must align with Solid Waste and Public Cleansing Management Act 2007, 2007, section 15 and MOH Guidelines 5.3. Sedekah redistribution with accredited charities is supported by Solid Waste and Public Cleansing Management Act 2007, 2007, section 32 and MOH Nutrition Guide 6.1. Successful implementation also requires adapting Thailand’s granular audits within Malaysia’s regulatory framework (Moorthy et al., 2024; Solid Waste and Public Cleansing Management Act 2007, 2007). A phased rollout – beginning with oncology wards, where waste peaks at 59.3% – will demonstrate ROI, reduce food costs and position private hospitals as sustainability leaders.

### Technology-enabled solutions framework

Intelligent waste management in Malaysian private healthcare hinges on integrating AI’s predictive portion control with blockchain’s immutable halal verification. Culturally-informed algorithms can incorporate kenduri and Ramadan dining patterns, while smart contracts ensure traceability throughout procurement to disposal (Razalli et al., 2021).

Implementation success requires upgrading network infrastructure, since only 40% of hospitals meet the ⩾50 Mbps threshold necessary for real-time processing. Targeted staff training must address digital-literacy gaps and vendor-support barriers identified in Malaysian healthcare contexts (Hira et al., 2022). Validation through 6-month pilot studies measuring waste reduction, cost-effectiveness and stakeholder acceptance provides essential empirical foundations before scaling decisions.

This phased, evidence-based approach balances technological innovation with cultural respect and establishes empirical foundations for scale-up. The successful implementation of technology-enabled solutions requires systematic policy frameworks that coordinate stakeholder efforts across multiple implementation phases.

### Policy implementation roadmap

The translation of empirical findings into actionable policy interventions requires systematic implementation approaches that acknowledge the complex interplay between regulatory frameworks, cultural considerations and organizational capacity constraints identified throughout this analysis. This roadmap establishes evidence-based priorities across short-, medium- and long-term implementation phases, ensuring alignment with existing regulatory requirements while building institutional capacity for sustainable waste management transformation within Malaysian private healthcare contexts.

Short-term priorities (0–12 months) focus on establishing foundational performance standards and validation frameworks. Setting plate-waste targets below 30% in routine audits creates accountability mechanisms while piloting culturally-adapted AI portion control in oncology and general wards enables algorithm refinement and efficacy validation. Mandatory halal segregation e-training aims to reduce ineligible surplus by 50%, addressing documented compliance gaps in waste segregation protocols.

Medium-term strategies (1–3 years) emphasize scaling validated interventions and integrating requirements into institutional frameworks. Deploying blockchain halal verification across supply chains targets 75% reductions in audit time, while embedding stream-specific waste audits into hospital accreditation criteria ensures systematic adoption. Matching grants for partnerships with pesantrens and religious councils incentivize collaborative waste-to-value initiatives that respect cultural and religious practices.

Long-term objectives (3–5 years) institutionalize comprehensive waste management frameworks and infrastructure capacity. Embedding socio-technical waste frameworks in national guidelines ensures systematic consideration of cultural-technical interactions, while MYR 50 million in IT and connectivity upgrades enables technology deployment across 80% of private hospitals. Mandatory annual reporting of waste metrics and sustainability outcomes promotes transparency and continuous improvement.

This phased roadmap aligns with the Solid Waste and Public Cleansing Management Act 2007 and MOH 2023 guidelines (Solid Waste and Public Cleansing Management Act 2007, 2007; Moorthy et al., 2024), leveraging regulatory levers and cultural values to drive sustainable waste reduction. These policy recommendations must be interpreted within the context of this review’s methodological strengths and limitations.

### Methodological considerations

This review’s strengths include dual-reviewer data extraction and comprehensive JBI quality appraisal, with high compliance in sampling adequacy (⩾85%) and statistical methods (⩾80%). Key limitations are outcome-assessor blinding (⩽55%) and confounder control (⩽60%), potential sources of bias. The English-only inclusion criterion and heterogeneous waste-measurement methods prevented meta-analysis; future studies should broaden language scope and standardize quantification protocols.

## Conclusions

This systematic review advances healthcare food waste management by integrating the TPB with socio-technical systems theory, establishing a culturally-adaptive framework that addresses the documented disconnect between sustainability awareness and persistent waste behaviours in Malaysian private healthcare. This theoretical integration demonstrates that effective interventions must simultaneously address individual behavioural drivers, organizational structures, and cultural-religious systems – moving beyond efficiency-focused approaches that fail in multicultural healthcare contexts.

The culturally-adaptive socio-technical framework provides three critical theoretical advancements. First, it positions cultural norms (kenduri hospitality traditions, halal compliance requirements) as structural determinants rather than peripheral considerations, explicating how subjective norms override individual sustainability intentions. Second, it demonstrates that technology adoption – including AI-driven portion control and blockchain halal verification – requires cultural calibration rather than direct transplantation from Western contexts. Third, it establishes that sustainable waste management in diverse healthcare settings depends on dynamic alignment between technological capabilities, organizational policies, and deeply embedded cultural-religious values.

Policy implications centre on phased implementation frameworks that embed cultural sensitivity within existing regulatory structures. The integration of stream-specific audits within the Solid Waste and Public Cleansing Management Act 2007 and MOH guidelines (Moorthy et al., 2024; Solid Waste and Public Cleansing Management Act 2007, 2007) demonstrates how policy levers can accommodate cultural practices while advancing sustainability targets. Comparative ASEAN analysis reveals that Malaysia’s multicultural healthcare context requires more nuanced policy approaches than those effective in culturally homogeneous systems, positioning Malaysian private healthcare to pioneer culturally-responsive sustainability frameworks with regional transferability.

Future research must validate this integrated framework through randomized controlled trials examining cultural-technology interactions, cost-effectiveness analyses across public-private contexts and mixed-methods studies exploring stakeholder acceptance. Standardized measurement protocols addressing current methodological heterogeneity will enable robust evidence synthesis and meta-analytic approaches.

This culturally-adaptive socio-technical framework represents a paradigm shift from efficiency-driven to values-integrated waste management, ensuring interventions respect cultural-religious practices while achieving measurable environmental and economic outcomes. Malaysia’s healthcare sector can lead regionally by demonstrating that sustainability and cultural preservation are complementary rather than competing objectives, establishing implementation models transferable across diverse ASEAN healthcare contexts.

## Future research priorities

Critical evidence gaps necessitate systematic research approaches addressing both methodological rigour and cultural sensitivity requirements. Twelve-month randomized controlled trials of culturally-adapted portion-control and audit systems in oncology and general wards must establish causal efficacy and long-term sustainability. Comparative cost-effectiveness analyses across private and public hospitals in Malaysia, Thailand and Indonesia will inform targeted policy investments and resource allocation decisions.

Sequential explanatory mixed-methods frameworks integrating quantitative surveys with in-depth interviews grounded in the TPB enable elucidation of patient and staff perceptions while refining implementation strategies so technological innovations align with sociocultural and religious values (Ahmad et al., 2023). Further development and application of socio-technical systems theory in conjunction with the TPB will capture the dynamic interplay of cultural, religious and technological drivers in healthcare food-waste management and support adaptive policy frameworks (Ahmad et al., 2023).

## Supplemental Material

sj-docx-1-wmr-10.1177_0734242X251408275 – Supplemental material for Food waste management in Malaysian private healthcare: A systematic review Supplemental material, sj-docx-1-wmr-10.1177_0734242X251408275 for Food waste management in Malaysian private healthcare: A systematic review by Maria Christina Barlet and Rashad Yazdanifard in Waste Management & Research

sj-docx-2-wmr-10.1177_0734242X251408275 – Supplemental material for Food waste management in Malaysian private healthcare: A systematic review Supplemental material, sj-docx-2-wmr-10.1177_0734242X251408275 for Food waste management in Malaysian private healthcare: A systematic review by Maria Christina Barlet and Rashad Yazdanifard in Waste Management & Research
