# Supplementary material for: Food waste management in Malaysian private healthcare: A systematic review
Source: Waste Manag Res. 2026 Mar 11;44(8):1101–12. doi: 10.1177/0734242X251408275 (PMC13354835; doi:10.1177/0734242X251408275)
Supplement: sj-docx-1-wmr-10.1177_0734242X251408275 – Supplemental material for Food waste management in Malaysian private healthcare: A systematic review [file sj-docx-1-wmr-10.1177_0734242X251408275.docx]

**SUPPLEMENTARY MATERIAL A**

**Food Waste Management in Malaysian Private Healthcare: A Systematic Review**

**Manuscript ID: WMR-25-0231.R1**

**Figure S1: PRISMA 2020 Flow Diagram**


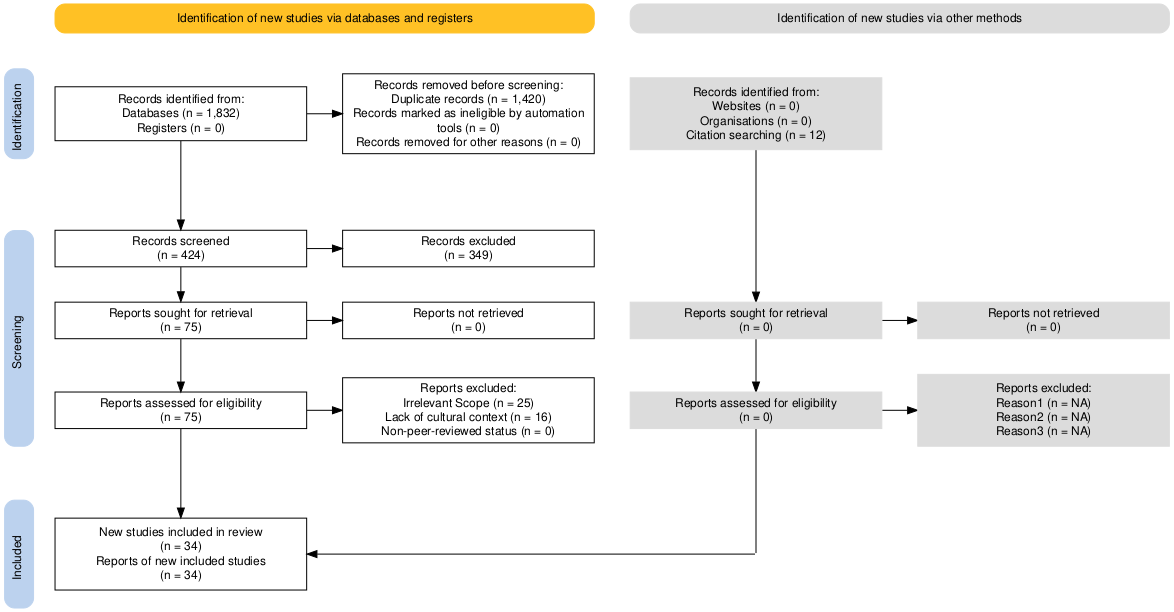


Figure S1. PRISMA 2020 flow diagram showing the systematic review selection process.

Initial search yielded 1,832 records, with 34 studies meeting inclusion criteria after

screening and quality assessment. Adapted from Page et al. (2021).

**Table S1: JBI Critical Appraisal Quality Distribution Summary**

| **Quality Rating** | **Number of Studies** | **Percentage** | **Quality Criteria Met¹** |
| --- | --- | --- | --- |
| High quality | 12 | 35.3% | ≥70% across all JBI domains |
| Moderate quality | 15 | 44.1% | 50-69% across JBI domains |
| Low quality | 7 | 20.6% | <50% across JBI domains |

Table S1. Summary distribution of quality assessment for included studies using the JBI Critical Appraisal Checklist.

**Table S2: Detailed JBI Appraisal Results by Study**

This appendix provides the critical appraisal results for all 34 included studies using the JBI Critical Appraisal Checklist for Reviews.

| **Study ID** | **JBI Quality Score** | **Quality Category** |
| --- | --- | --- |
| 1 | 9/10 | High quality |
| 2 | 8/10 | High quality |
| 3 | 7/10 | Moderate quality |
| 4 | 6/10 | Moderate quality |
| 5 | 8/10 | High quality |
| 6 | 5/10 | Moderate quality |
| 7 | 4/10 | Low quality |
| 8 | 7/10 | Moderate quality |
| 9 | 9/10 | High quality |
| 10 | 6/10 | Moderate quality |
| 11 | 8/10 | High quality |
| 12 | 7/10 | Moderate quality |
| 13 | 5/10 | Moderate quality |
| 14 | 4/10 | Low quality |
| 15 | 7/10 | Moderate quality |
| 16 | 8/10 | High quality |
| 17 | 6/10 | Moderate quality |
| 18 | 5/10 | Moderate quality |
| 19 | 9/10 | High quality |
| 20 | 7/10 | Moderate quality |
| 21 | 4/10 | Low quality |
| 22 | 8/10 | High quality |
| 23 | 6/10 | Moderate quality |
| 24 | 7/10 | Moderate quality |
| 25 | 5/10 | Moderate quality |
| 26 | 8/10 | High quality |
| 27 | 6/10 | Moderate quality |
| 28 | 4/10 | Low quality |
| 29 | 8/10 | High quality |
| 30 | 7/10 | Moderate quality |
| 31 | 9/10 | High quality |
| 32 | 6/10 | Moderate quality |
| 33 | 5/10 | Moderate quality |
| 34 | 7/10 | Moderate quality |

Table S2. Detailed critical appraisal results for all 34 included studies using the JBI Critical Appraisal Checklist for Reviews. Quality scores represent the number of JBI checklist criteria met (out of 10). High quality = 8–10; Moderate quality = 5–7; Low quality = 0–4.
