# Supplementary material for: Food waste management in Malaysian private healthcare: A systematic review
Source: Waste Manag Res. 2026 Mar 11;44(8):1101–12. doi: 10.1177/0734242X251408275 (PMC13354835; doi:10.1177/0734242X251408275)
Supplement: sj-docx-2-wmr-10.1177_0734242X251408275 – Supplemental material for Food waste management in Malaysian private healthcare: A systematic review [file sj-docx-2-wmr-10.1177_0734242X251408275.docx]

**Appendix A: JBI Appraisal Checklist Results**

This appendix provides the critical appraisal results for all 34 included studies using the JBI Critical Appraisal Checklist for Reviews.

| **Study ID** | **JBI Quality Score** | **Quality Category** |
| --- | --- | --- |
| 1 | 9/10 | High quality |
| 2 | 8/10 | High quality |
| 3 | 7/10 | Moderate quality |
| 4 | 6/10 | Moderate quality |
| 5 | 8/10 | High quality |
| 6 | 5/10 | Moderate quality |
| 7 | 4/10 | Low quality |
| 8 | 7/10 | Moderate quality |
| 9 | 9/10 | High quality |
| 10 | 6/10 | Moderate quality |
| 11 | 8/10 | High quality |
| 12 | 7/10 | Moderate quality |
| 13 | 5/10 | Moderate quality |
| 14 | 4/10 | Low quality |
| 15 | 7/10 | Moderate quality |
| 16 | 8/10 | High quality |
| 17 | 6/10 | Moderate quality |
| 18 | 5/10 | Moderate quality |
| 19 | 9/10 | High quality |
| 20 | 7/10 | Moderate quality |
| 21 | 4/10 | Low quality |
| 22 | 8/10 | High quality |
| 23 | 6/10 | Moderate quality |
| 24 | 7/10 | Moderate quality |
| 25 | 5/10 | Moderate quality |
| 26 | 8/10 | High quality |
| 27 | 6/10 | Moderate quality |
| 28 | 4/10 | Low quality |
| 29 | 8/10 | High quality |
| 30 | 7/10 | Moderate quality |
| 31 | 9/10 | High quality |
| 32 | 6/10 | Moderate quality |
| 33 | 5/10 | Moderate quality |
| 34 | 7/10 | Moderate quality |

*Note: Quality scores represent the number of JBI checklist criteria met (out of 10). High quality = 8–10; Moderate quality = 5–7; Low quality = 0–4.*
